# Supplementary material for: Critical Requirement of SOS1 for Development of BCR/ABL-Driven Chronic Myelogenous Leukemia
Source: Cancers (Basel). 2022 Aug 11;14(16):3893. doi: 10.3390/cancers14163893 (PMC9406065; doi:10.3390/cancers14163893)
Supplement: Supplementary file 1 [file cancers-14-03893-s001.zip › cancers-1848830-supplementary.pdf]

cancers MDPI

Supplementary Materials

Critical Requirement of SOS1 for Development of BCR/ABL-Driven Chronic Myelogenous Leukemia

Carmela Gómez [1,†], Rósula Garcia-Navas [1,†], Fernando C. Baltanás [1,2,3], Rocío Fuentes-Mateos [1], Alberto Fernández-Medarde [1], Nuria Calzada [1] and Eugenio Santos [1,*]

[1] Centro de Investigación del Cáncer, Instituto de Biología Molecular y Celular del Cáncer, CSIC-University of Salamanca and CIBERONC, 37007 Salamanca, Spain
[2] Instituto de Biomedicina de Sevilla (IBiS), Hospital Universitario Virgen del Rocío, CSIC, Universidad de Sevilla, 41013 Seville, Spain
[3] Departamento de Fisiología Médica y Biofísica, Universidad de Sevilla, 41013 Seville, Spain
* Correspondence: esantos@usal.es; Tel.: +34-923294801; Fax: +34-923294750
† These authors contributed equally to this work.

Supplementary Materials

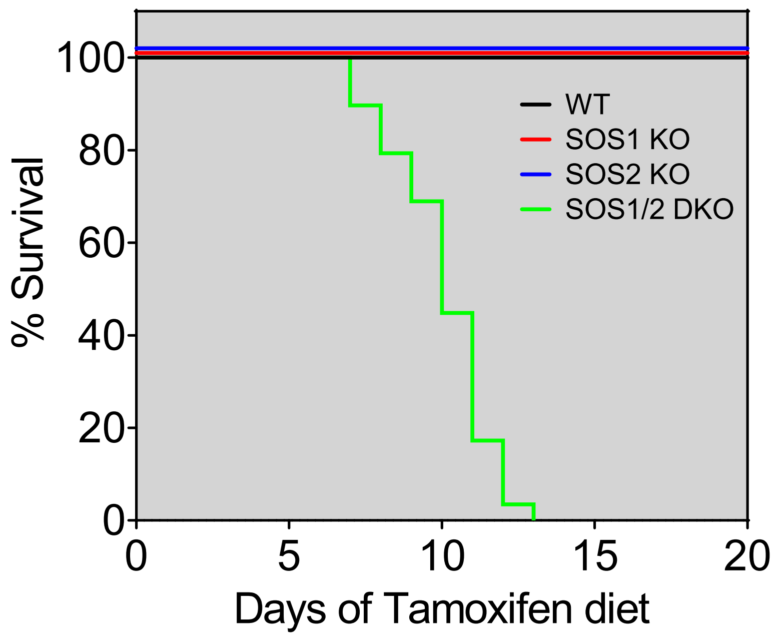

Figure S1. Kaplan-Meier survival plot showing that single SOS1 or SOS2 disruption did not compromise mouse survival in non-transgenic mice (black line) while SOS1/2 DKO mutants died 2 weeks after TAM-induced deletion of SOS1 (green line). n = 20 of WT, SOS1 KO, SOS2 KO and SOS1/2 DKO.

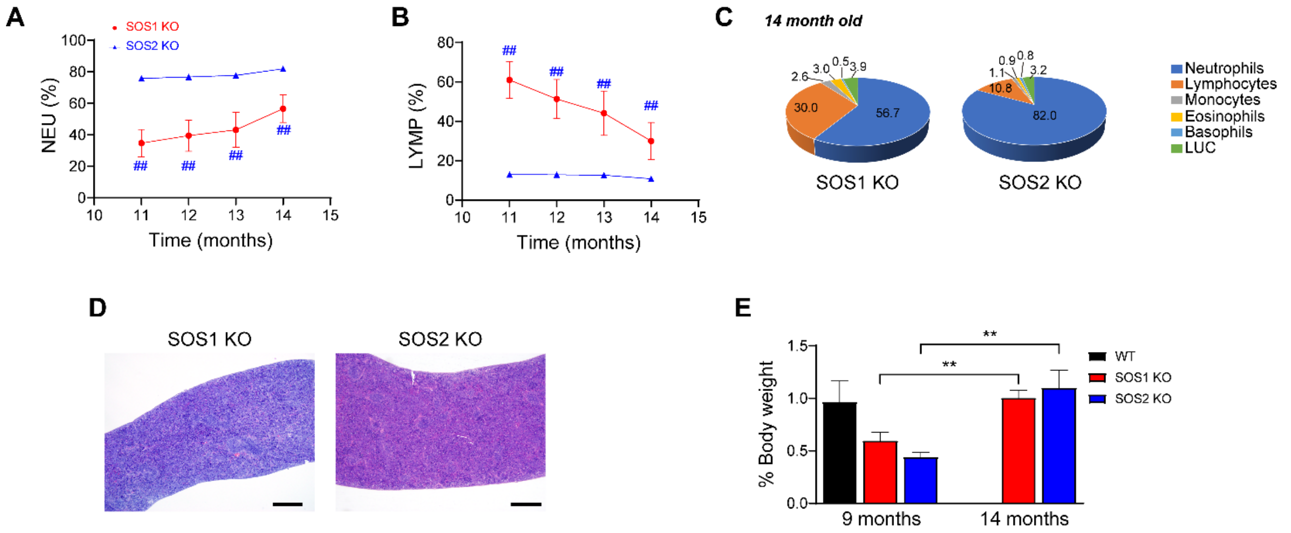

Figure S2. Kinetics of hematological parameters from peripheral blood of p210$^{BCR/ABL}$ transgenic mice of the indicated, color-coded SOS genotypes (SOS1 KO and SOS2 KO). The animals were treated with tamoxifen from 6 months of age to achieve inducible SOS1 ablation. The plots represent percentage of neutrophils (NEU) (**A**), and lymphocytes (LYMP) (**B**) in the peripheral blood of p210$^{BCR/ABL}$ transgenic mice. Values represented are mean ± s.e.m. n ≥ 4 for each genotype. # vs. SOS2 KO mice; ## $p < 0.01$. Percentage distribution of granulocytic and lymphocytic populations in peripheral blood of p210$^{BCR/ABL}$ transgenic mice of SOS1 KO and SOS2 KO at 14 months of age. The pie charts represent the percentage of neutrophils, lymphocytes, monocytes, eosinophils, basophils, and large unstained cells (LUC) (color-coded as indicated) in p210$^{BCR/ABL}$ transgenic mice (**C**). Hematoxylin and eosin (H&E) staining of histological sections of the spleen (**D**) 14-month-old p210$^{BCR/ABL}$ transgenic mice of the indicated genotypes. Spleen scale bar: 500μm. Bar plots representing the percentage of total body weight corresponding to the spleen (**E**) of p210$^{BCR/ABL}$ transgenic mice of the indicated (color coded) SOS genotypes (WT, SOS1 KO, and SOS2 KO) at 9 and 14 month-old. Values represented are the mean ± s.e.m. n≥3 for each genotype. **, $p < 0.01$.

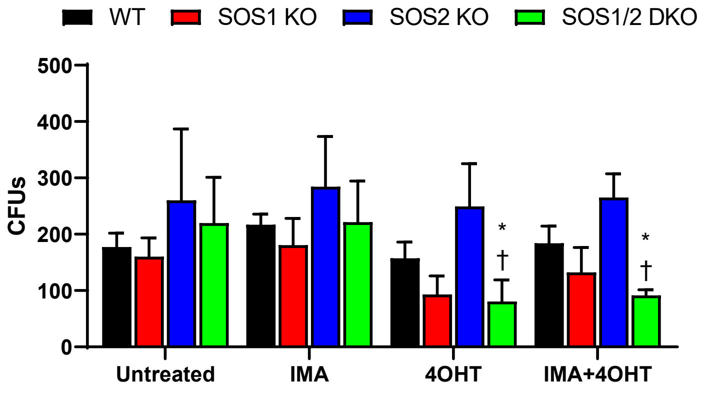

Figure S3. Analysis of the hematopoietic stem cell population from p210$^{BCR/ABL}$ transgenic mice of different SOS genotypes. Total numbers of colony forming units (CFUs) generated by the CD117+ cells isolated from the bone marrow of 9-month-old p210$^{BCR/ABL}$ mice of the indicated SOS genotypes (WT, SOS1 KO, SOS-KO and SOS1/2 DKO) after treatment of the cultures for 12 days with 4 hydroxy-Tamoxifen (4OHT, 2 μM), Imatinib (IMA, 3 μM) or both (4OHT+IMA), as indicated. Values represented the mean ± s.e.m. n≥3 for each genotype. * versus WT; † versus untreated; *, † $p < 0.05$.

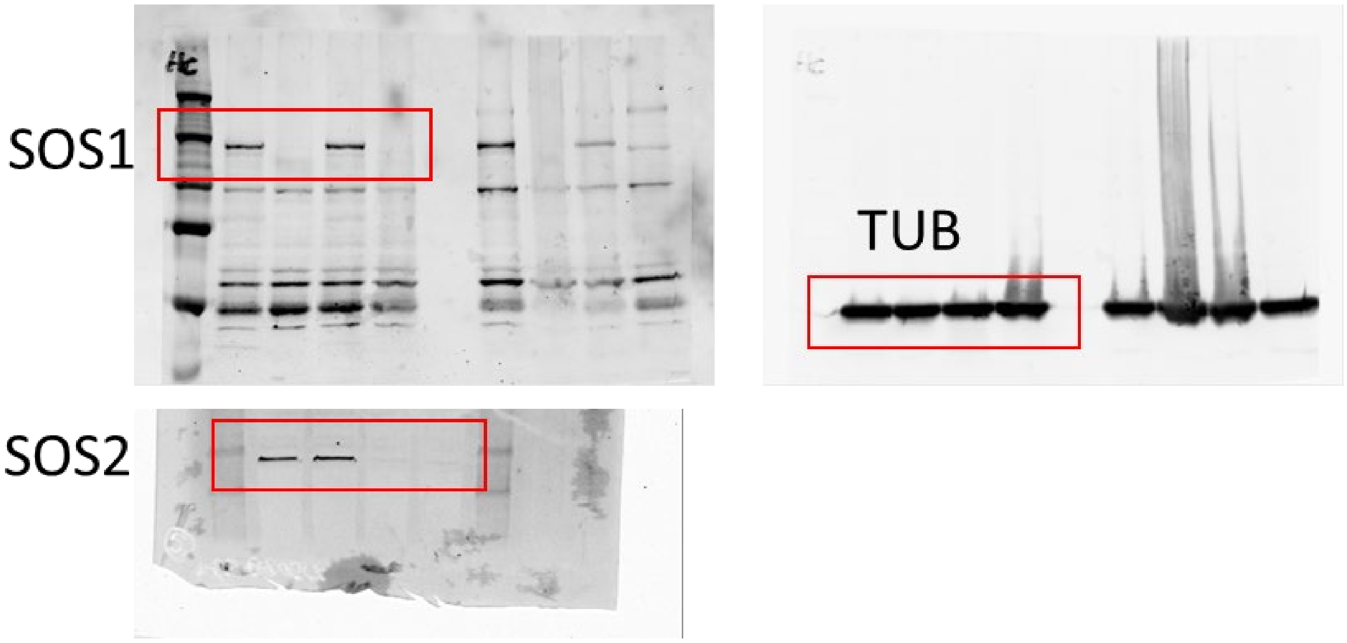

Figure S4. Uncropped blots shown in the Figure 1C of the manuscript.
